# Characterizing the Online Presence of Interventional Radiologists: A Potential Marketing Opportunity

**DOI:** 10.7759/cureus.9231

**Published:** 2020-07-16

**Authors:** Josh Bilello, Shaunak Patel, Vamsi Potluri, Gunvir S Gill, Arya N Bagherpour

**Affiliations:** 1 Interventional Radiology, University of Texas Medical Branch, Galveston, USA; 2 Radiology, University of Texas Medical Branch, Galveston, USA; 3 Medicine, University of Texas Medical Branch, Galveston, USA

**Keywords:** physician rating websites, online presence, social media, interventional radiology, radiology

## Abstract

Purpose

Patients increasingly utilize online resources to access healthcare information. Over the years, there has been an increasing trend of websites that allow patients to review their physicians. In many instances, the information found on these websites can be inaccurate or obsolete. This can affect patients' ability to make informed decisions about their provider choices. The need for interventional radiologists (IRs) is expected to rise due to an increasing demand for minimally invasive procedures. However, there is a lack of research regarding their online presence. Therefore, this study aims to characterize the online presence of IRs in the United States.

Materials

The Physicians Compare National Downloadable File (PCNDF) from the Center for Medicare Services was used to identify a sample of IRs in the United States. Then, a Google Custom Search Engine was created to parse the first ten search results for each physician using a set of search parameters. A coded script analyzed the URL contents of each link and placed the search results into one of the following categories: health or hospital system, third-party, social media, academic journal, or other.

Results

A total of 1,666 IRs were included for analysis. The results are as follows: 26.94% were from hospital or health systems, 66.93% were from third-party websites, 5.48% were from social media sites, 0.02% were from academic journals, and 0.64% were from other.

Conclusion

The online presence of IRs is primarily controlled by third-party websites, many of which do not allow physicians to manage their content. As the field of interventional radiology continues to grow; a great opportunity exists for physicians to expand their digital presence to more accurately reflect their practice.

## Introduction

Over the past two decades, the rapid growth of the Internet has enabled people to access a vast collection of knowledge. Potential patients often turn to the Internet to seek healthcare and provider information [1,2]. Simultaneously, physician rating websites (PRWs) have also been on the rise [3]. These websites are often controlled by third-parties and are not under the control of physicians or healthcare systems. However, patients often turn to these websites when making healthcare decisions or selecting providers [3,4]. PRWs provide a public forum for patients to review their own physicians and generally provide information regarding physician specialty training and location.

Interventional radiology continues to increase in demand as the field moves toward a more clinical model [5]. However, from a marketing standpoint “Interventional Radiology” is not an intuitive specialty name. Patients are unlikely to know the role intervention radiology plays in their health [6]. Previous reports suggest that other physicians incompletely understand the role and services interventional radiologist (IR) provides. This places an unnecessary burden on IRs' practices as they rely heavily on referrals from other physicians [7,8]. Therefore, an IR’s online presence can play a vital role in enabling patients to make informed decisions when selecting a provider. To date, the online presence of IRs has been minimally researched, making it difficult to measure the effectiveness of their digital footprint. This study aims to build on previous work by Bilello et al. to quantify and categorize the online presence of a sample of IRs participating in Medicare’s Fee for a Service program [9]. It was hypothesized that the online presence of IRs is primarily controlled by physician-rating websites instead of physicians.

## Materials and methods

This Health Insurance Portability and Accountability Act of 1996 (HIPAA) compliant study analyzed public physician information provided by Centers for Medicare and Medicaid Services (CMS) and was therefore exempted from review by an Institutional Review Board. The Physician Compare National Downloadable File (PCNDF) provided by CMS was used to collect data on a large sample of self-identified IRs. A total of 1,666 IRs were sampled in this study. National Provider Identifier (NPI) numbers were utilized to ensure the removal of any duplicates. Each IR’s first name, middle name, last name, gender, degree held, city, and state of service were recorded and were analyzed using Python Data Analysis Library (PANDAS) v0.25.0.

Due to the large number of physicians and the large amount of data to be collected, a Google custom search engine was developed to query the first ten Google search results for each IR in the PCNDF dataset. Each physician was queried using the following set of search parameters: “[First Name] + [Middle Name] + [Last Name] + [Interventional Radiology] + [City] + [State].” Ten search results were recorded for each physician which resulted in 16,666 unique URLs. These URLs were linked with the corresponding physician information provided by the PCNDF database. PANDAS was then used to export the table of physician information and URLs to a .csv file to be analyzed with Microsoft Excel later.

Each unique URL, 16,666 in total, was categorized into one of the five possible categories: PRWs, physician-controlled, social media, journal, or article, and others. The physician-controlled category also included hospital or healthcare system websites. Once all the 16,666 URLs were categorized, Excel formulas were used to determine how many links were in each category and which search result position they were in from one to ten. 

## Results

Of the 1,666 IRs included in this study, 1,642 had an M.D., 24 had a D.O. degree, 1,526 were male, and 140 were female. A total of 16,666 websites were indexed and categorized by this study. PRWs resulted in 10,433 (62.60%) of all search results. Physician-controlled sites comprised of 4,489 (26.94%) of all search results. Journal websites and articles comprised of only 4 (0.02%) of all search results. Websites labeled as other resulted in 106 (0.64%) of all search results. Lastly, social media sites made up 1,634 (9.80%) of all search results. Of the 1,634 social media sites, Doximity.com accounted for 721 of the results. Doximity is a social media website dedicated to physicians and other providers.

The most common website domain was www.healthcare4ppl.com, a website categorized as a PRW (Table 1). This site provides information on more than two million providers in the United States. Healthcare4ppl accounted for 1,394 unique URLs, making it 8.36% of all search results. The top ten URLs are displayed in Table 1 and show that most sites were controlled by a third-party website. No physician-controlled sites, social media sites, or academic journals were accounted for in the top ten site results.

**Table 1:** The top 10 websites that were indexed for a search on 1,666 IRs practicing in the United States. The first 10 results for each IR was indexed in this study (n = 16,666). PRW: physician rating website, IR: interventional radiologists.

Website Category	Website URL	Number of Times Indexed	% of all Searches Indexed
PRW	healthcare4ppl.com	1,394	8.36%
PRW	healthcare6.com	1,270	7.62%
PRW	caredash.com	1,187	7.12%
PRW	health.usnews.com	1,076	6.46%
PRW	npidb.org	1,013	6.08%
PRW	topnpi.com	947	5.68%
PRW	vitals.com	898	5.39%
PRW	doctor.webmd.com	841	5.05%
Social media website	doximity.com	721	4.33%
PRW	sharecare.com	675	4.05%
Total		10,022	60.13%

## Discussion

**Figure 1:**
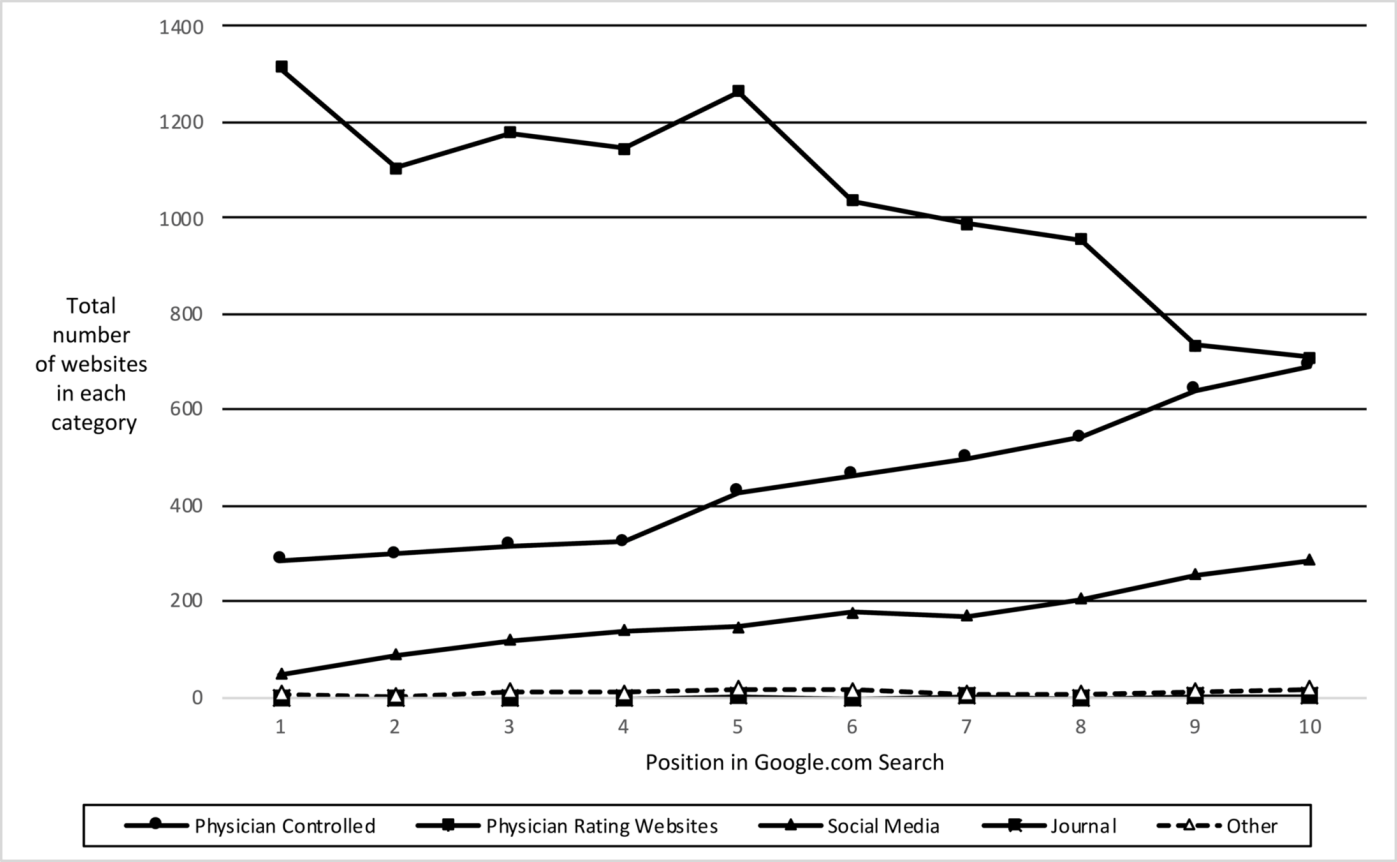
The frequency of each website category from a Google search of the top 10 results for 1,666 IRs practicing in the United States. The two largest categories are physician-controlled websites and PRWs. PRWs are most likely to be displayed first in a Google.com search. The probability of encountering a physician-controlled website increases almost linearly as you move further down the search index. PRWs: physician rating websites, IRs: interventional radiologists.

These results suggest that the online presence of IRs is overwhelmingly controlled by PRWs. PRWs account for 62.60% of all websites indexed while physician-controlled websites only account for 26.94%. Furthermore, physician review websites appear earlier on the search index than physician-controlled websites (Figure 1). A Pew study conducted in 2011 showed that 80% of internet users have looked online for information regarding healthcare [2]. Under this context, third-party control of IRs’ online presence is suboptimal for both patients and physicians due to potential misinformation.

Previous reports suggest that patients find PRWs useful when selecting physicians. One study reported 59% of patients considered PRWs as “somewhat important” or “very important” when selecting a physician [4]. However, PRW ratings are not correlated with clinical quality measures [10]. In fact, PRWs do not appear to correlate well with Press Ganey Surveys which are a validated metric of patient satisfaction [11]. Instead, PRWs focus on providing information regarding physicians’ qualifications, accepted insurances, practice location, and reviews from other patients. Furthermore, PRWs typically offer little to no clinical information for patients.

Historically speaking, PRWs have focused on adding data from primary-care physicians, medical subspecialists, and surgeons. In 2015, Gilbert et al. reported that only 19.7% of 1,000 randomly selected diagnostic radiologists were cataloged on any of the five main PRWs [12]. It has also been reported that radiologists, anesthesiologists, and pathologists have the lowest likelihood (6.6%) of receiving a patient review even when those specialists are profiled on PRWs [13]. Pairing these circumstances with IR’s recent emergence as a primary specialty creates an opportunity for IRs to control and increase their online presence as patients continue to utilize the Internet as a tool in regards to their healthcare decisions.

Recommendations

1. Utilize Social Media

The results of this paper show that social media websites for IR’s only account for 9.8% of all search results. However, nearly half of the social media profiles created by IRs are on Doximity.com which is a social media website dedicated to healthcare professionals which does not allow patients to have meaningful access to the platform. Popular social media websites that patients do have access to include Facebook, Twitter, Instagram, and Snapchat. Hage et al. report that tweets related to IR have more than quadrupled over a three year period [14]. Other specialties have long embraced social media for the purposes of patient outreach, education, and advertising [15,16]. While social media can be used as a tool to connect with others, IRs and other medical providers should receive training on how to utilize this media without breaking HIPAA barriers before creating their respective pages.

2. Create a Website to Further Develop an Online Presence for Your Practice or Institution

It is expected that physician-reimbursement models will soon account for patient satisfaction [17,18]. In several ways, a website can increase communication between physicians and patients. First, a practice website can be used to deliver test results to patients in a timely manner. Second, a website can be used to deliver quality healthcare information to patients to help them make well-informed decisions [19]. Furthermore, a well-designed website will also function as a form of marketing for services offered. Several strategies exist to increase traffic to a practice website but are outside the scope of this paper.

3. Network Outside of the Internet

A strong online presence can be supported by a strong physical presence too. IRs should seek to develop stronger ties with referring physicians and the community at large. Other forms of advertisement may also generate significant referrals, such as radio advertisements [20]. Another way to build a strong network is to assume a leadership position within a hospital network. These alternative strategies can be used to promote the practice website and services offered.

## Conclusions

The results of this study may be generalizable as the 1,666 IRs sampled represent approximately 39.3% of the expected IR workforce. Overall, this study found that the online presence of IRs is controlled overwhelmingly by PRWs that are controlled by third parties. Current literature suggests that PRWs are not correlated to patient outcomes or patient satisfaction. This creates an opportunity for potential misinformation or misunderstanding to be spread. Therefore, it is vital for IRs to boost their professional identity online so that patients can receive the most up to date information about their physician and services provided. Furthermore, for IRs who practice in a clinic-based outpatient setting, the Internet can be a powerful tool for them to connect and engage with patients and other physicians for practice building.
